# Mechanism of ribosome stalling by the AMD1 C-terminal tail arrest peptide

**DOI:** 10.1126/sciadv.aec5067

**Published:** 2026-03-27

**Authors:** Emir Maldosevic, Fabio S. Boiocchi, Michal I. Swirski, Kyle A. Meiklejohn, Martina M. Yordanova, Pavel V. Baranov, Ahmad Jomaa

**Affiliations:** ^1^ Department of Molecular Physiology and Biological Physics, University of Virginia, Charlottesville, VA 22903, USA.; ^2^ School of Biochemistry and Cell Biology, University College Cork, Cork T12 K8AF, Ireland.; ^3^ Institute of Genetics and Biotechnology, Faculty of Biology, University of Warsaw, 02-106 Warsaw, Poland.; ^4^ Center for Membrane and Cell Physiology, University of Virginia, Charlottesville, VA 22903, USA.

## Abstract

*AMD1* encodes adenosylmethionine decarboxylase 1 (AMD1), a key enzyme in polyamine biosynthesis. A subset of ribosomes translating the *AMD1* coding sequence read through the stop codon and pause at a second in-frame stop 384 nucleotides downstream, producing a conserved C-terminal extension (C-tail). Despite growing evidence that such cis-acting elements regulate translation of their genes, the molecular mechanism by which the C-tail mediates ribosome stalling remains unclear. Here, we determined the structure of the ribosome nascent chain complex paused by the AMD1 C-tail which traps eukaryotic release factor 1 (eRF1) with the ATP-binding cassette subfamily E member 1 (ABCE1). The nascent chain forms a molecular clamp that positions an arginine hook in the peptidyl-transferase center, occluding the accommodation of the eRF1 GGQ motif thereby hampering translation termination. Analysis of aggregated ribosome profiling data revealed several genes with a pattern of stop codon readthrough followed by ribosome stalling at a specific location, suggesting that regulatory readthrough-stall mechanisms may not be limited to *AMD1*.

## Teaser

An arrest peptide encoded within the *AMD1* 3′ UTR locks the ribosomal PTC to evade translation termination.

## INTRODUCTION

Cells respond to environmental cues and shifting metabolic demands by regulating gene expression to adapt and ensure survival. Regulation can occur at the level of protein synthesis during different steps of translation, including elongation and termination (*1*–*10*). Emerging evidence indicates that ribosomes actively control protein synthesis by sensing elements encoded in cellular mRNAs and nascent chains as they pass through the mRNA channel or polypeptide exit tunnel, respectively. Programmed ribosome pausing along these sequence elements modulates gene expression (*11*–*14*), regulates metabolism (*15*–*18*), and enables cells to overcome intracellular stress (*19*–*22*).

Polyamines are ubiquitous polycationic molecules that bind to negatively charged nucleic acids, regulate their metabolism, and are essential for cell growth and proliferation (*23*–*25*). At the level of protein synthesis, polyamines regulate translation efficiency and fidelity (*26*, *27*). Intriguingly, nascent peptides encoded by key polyamine metabolism enzyme transcripts have emerged with a role in sensing polyamine levels from within the ribosomal tunnel, enabling mechanisms such as ribosome stalling (*16*, *28*, *29*) and frameshifting (*30*, *31*).

Adenosylmethionine decarboxylase 1 (AMD1; also known as AdoMetDC), is one such enzyme essential for both spermine and spermidine biosynthesis (*32*). The conserved stalling peptide, MAGDIS, encoded by a regulatory translon (*33*) located close to the 5′ end of the *AMD1* mRNA controls AMD1 enzyme levels depending on intracellular spermidine concentrations (*28*, *34*, *35*). Under elevated spermidine conditions, ribosomes pause at the stop codon of the MAGDIS-encoding translon. The paused ribosome would then sterically block assembly of preinitiation complexes at *AMD1* mRNA preventing translation initiation at the downstream start codon in the *AMD1* coding sequence (CDS), thereby reducing AMD1 synthesis.

A recent study used ribosome profiling to identify a second highly conserved stalling site downstream of *AMD1* CDS (*36*). During translation of the CDS, a small subset of ribosomes read through the stop codon (*36*, *37*) and synthesize a 127–amino acid–long C-terminal extension (C-tail). Ribosomes will then pause at a downstream in-frame stop codon, exposing the AMD1 C-tail while it remains tethered to the ribosome. Addition of this C-tail leads to a reduction in the levels of the extended protein (*36*, *38*). Despite growing evidence that cis-acting elements regulate translation of their gene (*1*, *2*, *14*), the precise regulatory elements used by the AMD1 C-tail remain unclear. Furthermore, it is not understood why nascent chains fail to release from ribosomes stalled at a stop codon of the AMD1 C-tail extension, suggesting that translation termination is impaired.

## RESULTS

### eRF1 and ABCE1 are trapped on ribosomes translating the AMD1 C-tail

**Fig. 1.**
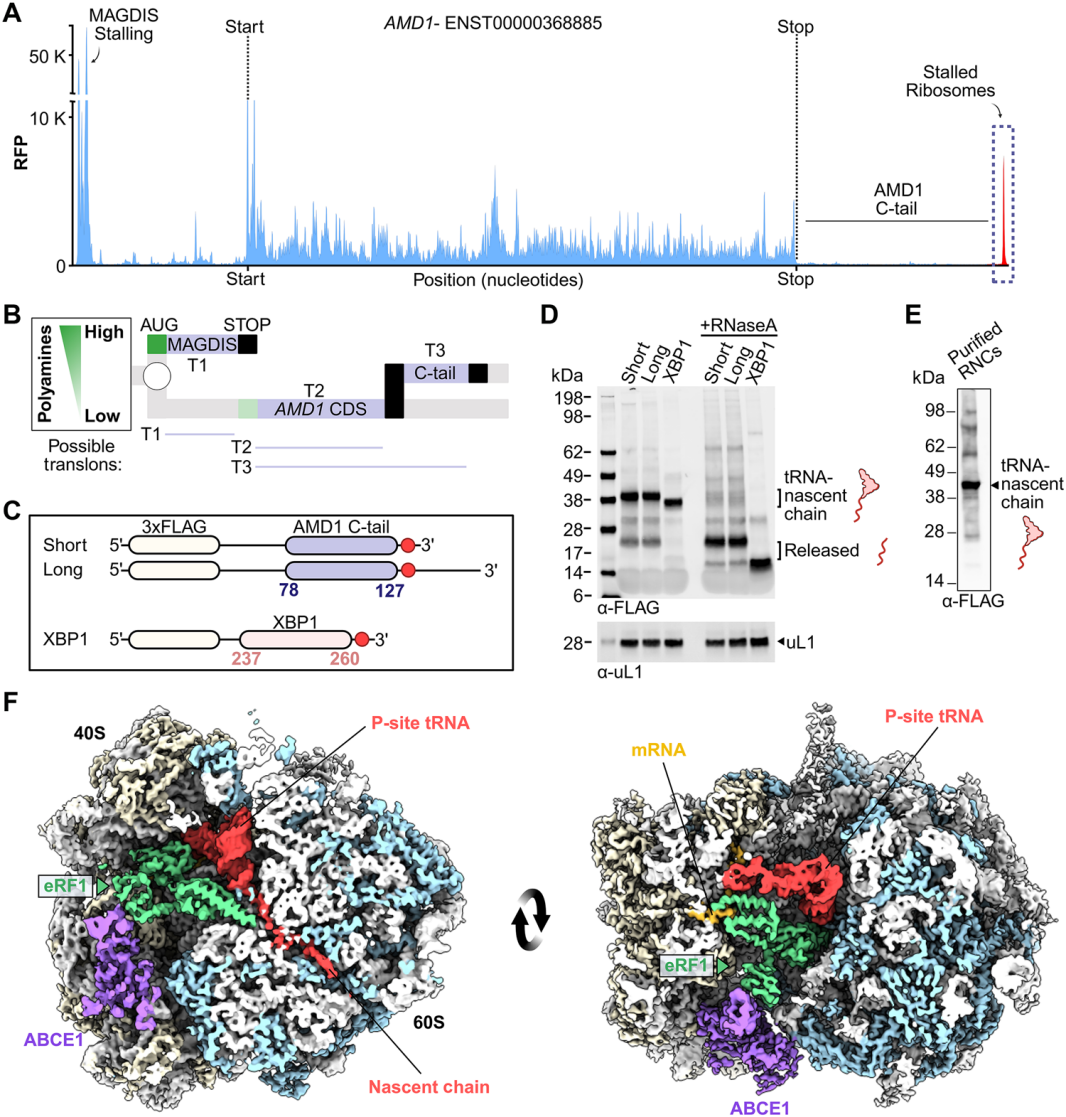
Cryo-EM structure of ribosomes stalled on the 3′ UTR of the *AMD1* mRNA. (**A**) Ribosome profile of the *AMD1* transcript highlighting the observed ribosome pausing events in the 5′ and 3′ UTRs generated with ribocrypt.org. (**B**) Ribosome decision graph showing translons (T1, T2, and T3) organization of the *AMD1* mRNA illustrating polyamine-dependent control of *AMD1* CDS translation. (**C**) Schematic diagram for the mRNA used to stall ribosomes with the AMD1 C-tail (AAs 78 to 127) and with XBP1 (AAs 237 to 260) as a control. Red circle indicates the position of the stop codon. (**D**) In vitro translation reactions of the indicated mRNAs from (C). (**E**) Western blot of purified ribosomes stalled at the C-tail of AMD1. (**F**) Cryo-EM density map of the ternary complex filtered to 4 Å for visualization. Cross sections of the map shows that the P-site tRNA and nascent chain remain attached to ribosomes with ABCE1 and eRF1 bound. Colors: gray-rRNA; blue, large subunit r-proteins; beige, small subunit r-proteins; yellow, mRNA; purple, ABCE1; green, eRF1; red, tRNA-NC.

Analysis of publicly available ribosome profiling data in the *AMD1* locus using RiboSeq.Org tools (*39*) showed peaks of high ribosome density both upstream and downstream of the *AMD1* CDS (*36*). A prominent peak, 384 nucleotides downstream of the CDS, corresponds to ribosomes stalled at a stop codon following the translation of the AMD1 C-tail (“stalled ribosomes;” Fig. 1, A and B). As the numbers of vertebrates with available ribosome profiling data have increased since the original publication (*36*), we have expanded the analysis to these species. Peaks of ribosomal footprints corresponding to the stalling location in human *AMD1* mRNA are evident in most species with available data despite varying coverage and quality of the data (fig. S1, A and B). This suggests that not only the C-tail extension but also ribosome stalling mediated by it are universally conserved across vertebrates.

To reconstitute this pausing event, we generated constructs encoding the C-tail of AMD1 fused to an N-terminal 3X-FLAG via a flexible linker and monitored ribosome stalling using an in vitro translation system in rabbit reticulocyte lysates (Fig. 1, C and D). Immunoblot analysis demonstrated that ribosomes were stalled with a nascent chain still bound to a P-site transfer RNA (tRNA), as indicated by a single band at ~45 kDa, the combined molecular weights of the nascent chain (20 kDa) and tRNA (25 kDa). Treating the samples with ribonuclease A (RNaseA) released nascent chains from the tRNA indicated by a shift in the nascent chain band size to ~20 kDa (Fig. 1D). Further extending the mRNA construct by an additional 135 bases of the native downstream sequence after the C-tail stop codon did not shift the molecular weight of the stalled product when compared to the shorter construct (Fig. 1D). These results indicate that pausing occurs at the stop codon of the *AMD1* extended translon without the release of the nascent chain and independent of RNA sequence downstream of the stop codon, consistent with previous results (*36*).

Next, we purified the AMD1 C-tail ribosome nascent chain complex (RNC_AMD1C_) and determined its structure using cryogenic electron microscopy (cryo-EM) (Fig. 1, E and F). An initial two-dimensional (2D) classification followed by a subsequent 3D classification of the particles was conducted to remove nonribosomal or heterogeneous particles in different translation states (fig. S2A). This approach yielded a cryo-EM map of the stalled 80S ribosome that displayed strong density for the P-site tRNA and a nascent chain present in the polypeptide exit tunnel (fig. S2A, red). The density of the nascent chain can be unambiguously assigned to the AMD1 arresting peptide tethered to the P-site tRNA within the ribosome. Furthermore, two additional bound factors were observed at the interface of the ribosomal subunits (40S and 60S) (fig. S2, B and C, purple). One of the bound factors is the release factor eRF1 resolved interacting with mRNA in the A-site (Fig. 1F and fig. S3, A to D, green and yellow). The second density corresponded to the recycling factor ABCE1 (Fig. 1F and fig. S3E, purple) and together these components form the ternary ABCE1:eRF1:RNC_AMD1C_ complex (movie S1 and table S1).

### The GGQ motif of eRF1 is sequestered in an inactive conformation

**Fig. 2.**
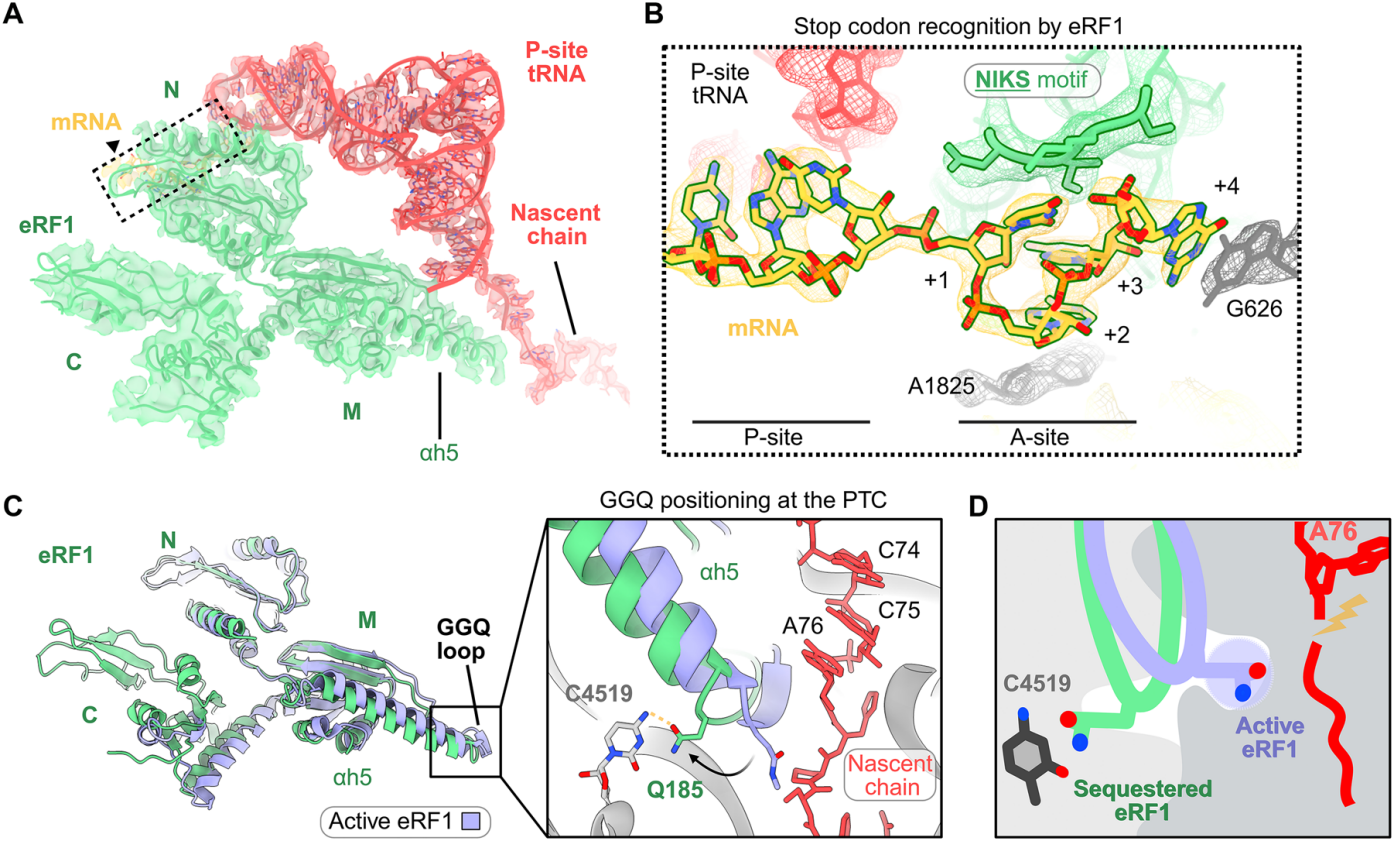
eRF1 adopts an inactive conformation on ribosomes stalled on the AMD1 C-tail. (**A**) Overview of eRF1 domains involved in stop codon recognition (N-domain) and facilitating nascent peptide hydrolysis (M-domain). The sharpened EM map (b-factor of −29.0 Å^2^) was used to visualize the N- and M-domains of eRF1 as well as the tRNA-nascent chain, while the flexible C-domain of eRF1 was visualized using a 4-Å low-pass filtered map. (**B**) Close-up of the boxed region in (A) depicting the compaction of the stop codon in the A-site following recognition by the eRF1 N-domain. (**C**) Comparison of eRF1 accommodation to a previous termination complex of active eRF1 (PDB 6XA1). Inset shows a close-up of the conformational changes in the GGQ loop relative to active eRF1. (**D**) Schematic model depicting the proper positioning requirement of Gln^185^ for peptide hydrolysis.

Translation termination is a multistep process that requires stop codon recognition and hydrolysis of the peptidyl-tRNA ester bond to release nascent proteins from the ribosome (*40*, *41*). Initially, eRF1 is delivered to the ribosome as a complex with eRF3•guanosine 5′-triphosphate (GTP). Upon stop codon recognition, GTP hydrolysis triggers dissociation of eRF3 from eRF1, allowing eRF1 to fully accommodate into the A-site (*41*–*45*). The N-domain of eRF1 recognizes stop codons in the A-site of the ribosome (Fig. 2A, “N”), while its M-domain (Fig. 2A, “M”) extends toward the peptidyl transferase center (PTC) to promote peptide hydrolysis (*6*, *44*–*46*). The M-domain contains a universally conserved GGQ motif located in a flexible loop adjacent to α-helix 5 (Fig. 2A, “αh5”), which is used to facilitate the hydrolysis reaction via a methylated Gln^185^ residue (*47*–*49*). Previous studies have shown that ABCE1 copurifies with eRF1 on ribosomes, indicating that eRF1 serves as a platform for ABCE1 binding (*45*, *46*). This enables ABCE1 to split and recycle ribosomes immediately following termination, consistent with its known function (*50*, *51*).

Here, both eRF1 and ABCE1 natively copurified with ribosomes paused while translating the AMD1 C-tail (Fig. 1F). The N- and M-domains of eRF1 were positioned in the A-site and resolved at 2.5 to 3.5 Å (figs. S2C and S3, A to D). The conserved NIKS, YxCxxxF, and GTS motifs in the eRF1 N-domain engage the A-site mRNA, and together with Gln^55^, coordinate stop codon compaction and recognition as previously described (Fig. 2B and fig. S4A) (*6*, *45*, *46*, *49*). A comparison of our structure to prior eRF1-ribosome complexes showed that the stop codon adopts the canonical architecture in the A-site following recognition (fig. S4B) (*45*, *46*, *49*). Notably, mutating the native stop codon to alternative stop codons (UAA/UGA) or a sense codon (GCG) did not affect ribosome stalling (fig. S4C), suggesting that stalling by the C-tail must occur before eRF1 binding to prevent termination.

To determine how eRF1-mediated termination and therefore nascent chain release was blocked in the ternary ABCE1:eRF1:RNC_AMD1C_ complex, we compared our structure to a previously published termination complex (*49*). Both αh5 and the loop containing the GGQ motif undergo a conformational change which sequesters Gln^185^ ~8 Å away from the position it adopts during translation termination, in a small pocket formed by ribosomal RNA (rRNA) bases C4399, U4398, C4519, and C4453 (Fig. 2C and fig. S5, A and B). This conformation of GGQ motif is different from recent structures of termination complexes reported from both eukaryotes and bacteria where the GGQ motif of either eRF1 or bacterial RF1 was positioned near the nascent chain to coordinate hydrolysis at the PTC (fig. S5, B and C) (*49*, *52*). Furthermore, in our structure the P-site loop of uL16 adopts a distinct conformation that contacts the tRNA, while eRF1 Phe190 stacks with A4548, which differs from the conformation observed in the active eRF1 complex (fig. S6, A and B). Together, these observations show that eRF1 is sequestered and stabilized in an inactive state on the ribosome (Fig. 2D) mediated by the AMD1 C-tail arresting peptide.

### A molecular clamp orchestrates AMD1 C-tail compaction in the PET

**Fig. 3.**
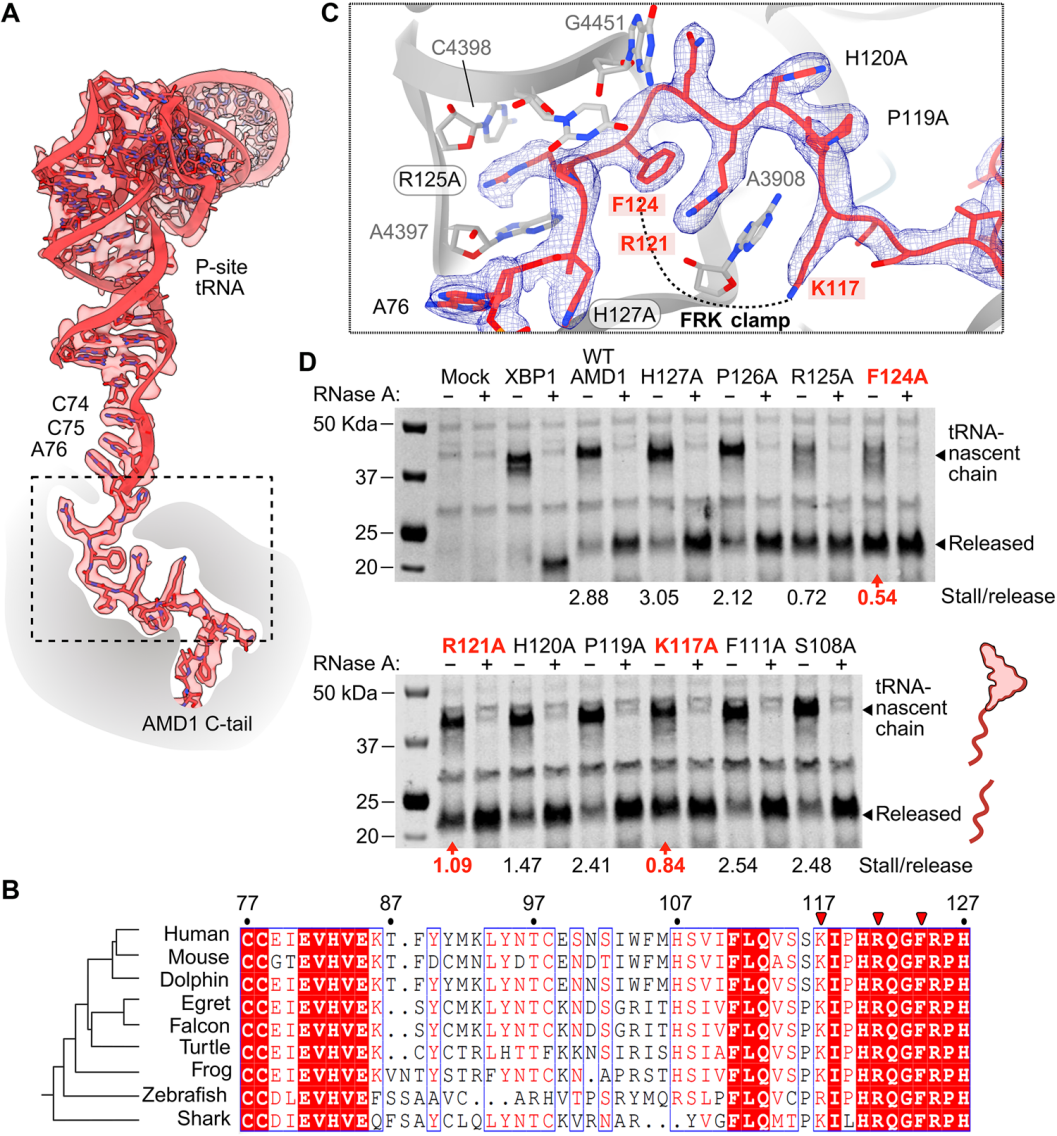
A conserved molecular clamp in the C-tail arresting peptide mediates ribosome stalling within the PET. (**A**) Overview of the tRNA-nascent chain conformation showing the sharpened EM map (b-factor of −29.0 Å^2^) as a transparent surface. (**B**) Sequence alignment of the AMD1 C-tail among representative vertebrates. Red arrowheads indicate the clamp residues depicted in (C). (**C**) Close-up of the early stacking interactions and molecular clamp of the AMD1 C-tail with the ribosome. The sharpened map is shown as a blue mesh. (**D**) Ala scanning mutagenesis experiment identifying residues contributing to ribosome stalling. Band intensities were quantified, and the ratio of stalled nascent chain (tRNA-nascent chain) to released nascent chain was calculated for each lane (stall/release). Clamp mutants are shown in red.

To investigate how the C-tail is driving ribosome stalling, we analyzed its interactions with the ribosome (summarized in fig. S7A). Several turns formed by the nascent peptide, position highly conserved amino acid side chains to interact with rRNA bases that line the PET (Fig. 3, A to C). Notably, Phe^124^, Arg^121^, and Lys^117^ (FRK clamp) capture the rRNA base A3908 by forming a series of π-stacking interactions (Fig. 3C, “dashed line,” and movie S2). This clamp stabilizes the nascent chain in the tunnel before it is looped back toward the constriction site formed by uL22 and uL4. As a result, the nascent peptide adopts a unique Z-shaped configuration within the PET, accommodating an additional six amino acids in the tunnel compared with the path of a nascent peptide that does not arrest translation (fig. S8, A and B) (*53*). Furthermore, both the conformation and interactions of the stalling peptide are distinct from the structures of previously reported stalling peptides such as XBP1 (*3*) and the helical hCMV mammalian arresting peptides (*6*) (fig. S8, C and D).

Sequence alignments revealed that the amino acids involved in the observed ribosome contacts were highly conserved across vertebrates (Fig. 3B). To test whether these residues were crucial for AMD1 stalling, we conducted an alanine scanning mutagenesis screen in rabbit reticulocyte lysates and monitored ribosome stalling for each alanine mutant relative to wild-type (WT) AMD1. Mutations that disrupt stalling promote nascent chain release, which is detected by Western blotting as an accumulation of a lower–molecular weight released product accompanied by a corresponding decrease in the higher–molecular weight peptidyl-tRNA. Using this approach, we found that FRK clamp mutations decreased the stall-to-release ratio by approximately three- to fivefold relative to the WT AMD1 C-tail, as indicated by the increased levels of released nascent chain (Fig. 3D). In contrast, mutations before and after the FRK clamp had a more subtle effect on stalling, as indicated by reduced release of the nascent chain from the tRNA and thus contribute less to ribosome stalling.

### The AMD1 C-tail inserts an arginine hook into the PTC to block translation termination

**Fig. 4.**
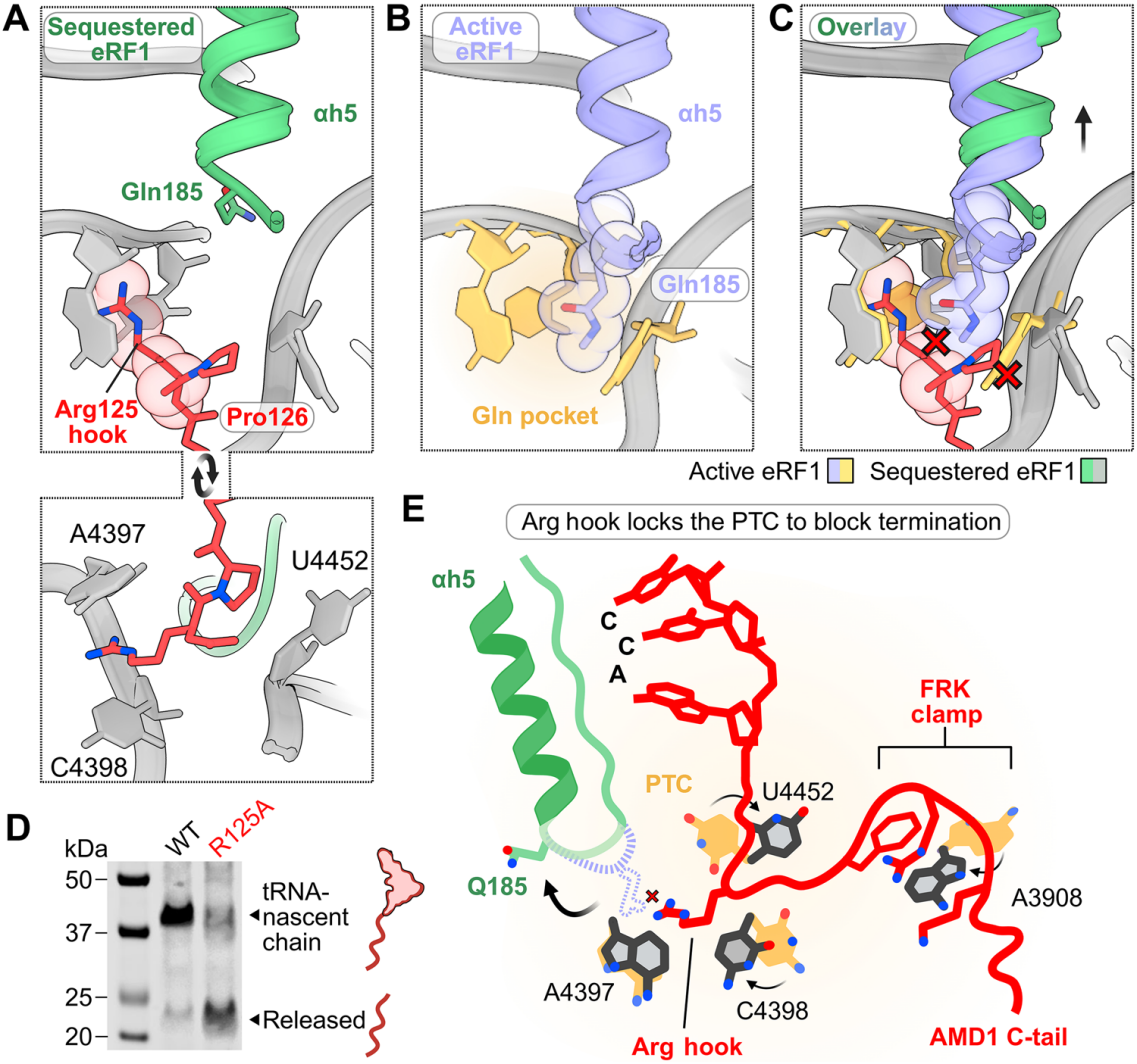
Insertion of an Arg hook into the PTC mediates termination inhibition by the AMD1 C-tail. (**A**) Insertion of the AMD1 C-tail Arg^125^ side chain into the PTC. (**B**) Proper positioning of the GGQ loop in the active eRF1 complex (PDB 6XA1). PTC residues A4397, C4398, and U4452, which coordinate positioning of Gln^185^ are colored yellow. (**C**) Overlay showing the steric clash of the Arg hook with Gln^185^ from the active eRF1 complex. Steric clashes are depicted by a red X. (**D**) In vitro translation reaction monitoring ribosome stalling when Arg^125^ is mutated to Ala. (**E**) Schematic model for the mechanism of termination inhibition by the AMD1 C-tail.

Given that the FRK clamp lies ~15 to 20 Å from the peptidyl-tRNA ester bond where hydrolysis occurs, it is unclear how and whether this conformation contributes to termination inhibition. To determine specifically how translation termination was blocked and what leads to eRF1 GGQ motif sequestration away from the PTC, we closely analyzed conformational changes in the PTC triggered by the AMD1 C-tail stalling peptide. Notably, we found that the highly conserved Arg^125^ side chain of the arrest peptide inserts into the PTC, between rRNA bases A4397 and A4398 (Fig. 4A). In particular, this Arg “hook-like” insert remodels the surrounding rRNA bases and sterically occludes the accommodation of eRF1 Gln^185^, preventing it from reaching the peptidyl-tRNA ester bond where nascent chain hydrolysis occurs (Fig. 4, B and C, and movie S3). Furthermore, we show that mutation of the Arg hook alone, to a nondisruptive shorter Ala side chain, leads to nascent chain release (Fig. 4D). Together, these results reveal that ribosome stalling on the extended *AMD1* translon is driven by a conserved FRK clamp, which then inserts an Arg hook into the PTC to block eRF1 GGQ accommodation and inhibit translation termination (Fig. 4E).

### Ribosome stalling downstream of CDS in other genes

Given that AMD1 C-tail-mediated stalling occurs downstream of the CDS within the *AMD1* 3′ untranslated region (UTR), we next analyzed available ribosome profiling datasets to determine whether analogous stalling sites could be detected within 3′ UTRs of other mRNAs with evident stop codon readthrough. The number of available ribosome profiling samples has more than tripled since the discovery of AMD1 C-tail stalling (*39*). Aggregating these data greatly improves signal to noise ratio in ribosome profiling data analyses. Using this updated aggregated dataset, we manually reexamined the list of genes with high ribosome profiling density downstream of annotated CDS reported in Yordanova *et al.* (*36*). For the majority of reported cases, ribosome footprint density downstream of CDS results from overlaps with translons on other transcripts. Only a few cases (e.g. *CENPA*, *CENPB*, *EEF1A2*, *GPX1*, *MACROD2*, and *ORMDL3*) showed confident evidence of high ribosomal footprint densities likely originating from the same transcript.

To extend the search, we have decided to look specifically at genes with evidence of stop codon readthrough (see Materials and Methods). After excluding cases where the peaks may have arisen due to ambiguous mapping of sequencing reads potentially originating from different loci, we identified increased peaks of density in 12 genes: *AGPAT4*, *AMD1*, *BRI3BP*, *CITED2*, *CPNE8*, *ETNPPL*, *KLC4*, *LDHB*, *MAPK10*, *ORMDL3*, *SACM1L*, and *TENT5B* (representative examples shown in fig. S9, A to D). We found similar ribosome peaks in the orthologs of three genes, i.e., *EEF1A2*, *MAPK10*, and *SACM1L* (fig. S10, A to C). Furthermore, visual examination of nucleotide conservation using a phyloP track constructed for 100-way vertebrate genomes (*54*) reveals higher conservation upstream of these stalling sites. In addition, *MAPK10* and *SACML1* have been previously reported to have conserved readthrough regions that exhibit codon substitution patterns in multiple genomic sequence alignments, typical for protein coding genes, as measured with PhyloCSF (*55*). This suggests evolutionary selection acting on the sequences encoding nascent peptides that stall in the ribosome exit tunnel during translation, which would occur if stalling in these genes confers biological functions positively affecting Darwinian fitness. Given the sequence conservation and occurrence of ribosome stalling, it is very likely that stalling is functional not only in *AMD1* but also in case of these three genes.

## DISCUSSION

There is growing evidence that cis-acting elements affect translation of their own genes. *AMD1* is one such gene whose expression levels are proposed to be regulated by ribosome stalling in both its 5′ leader and 3′ trailer (*28*, *36*). However, the molecular mechanism by which the C-tail induces ribosome stalling hitherto remained uncharacterized.

**Fig. 5.**
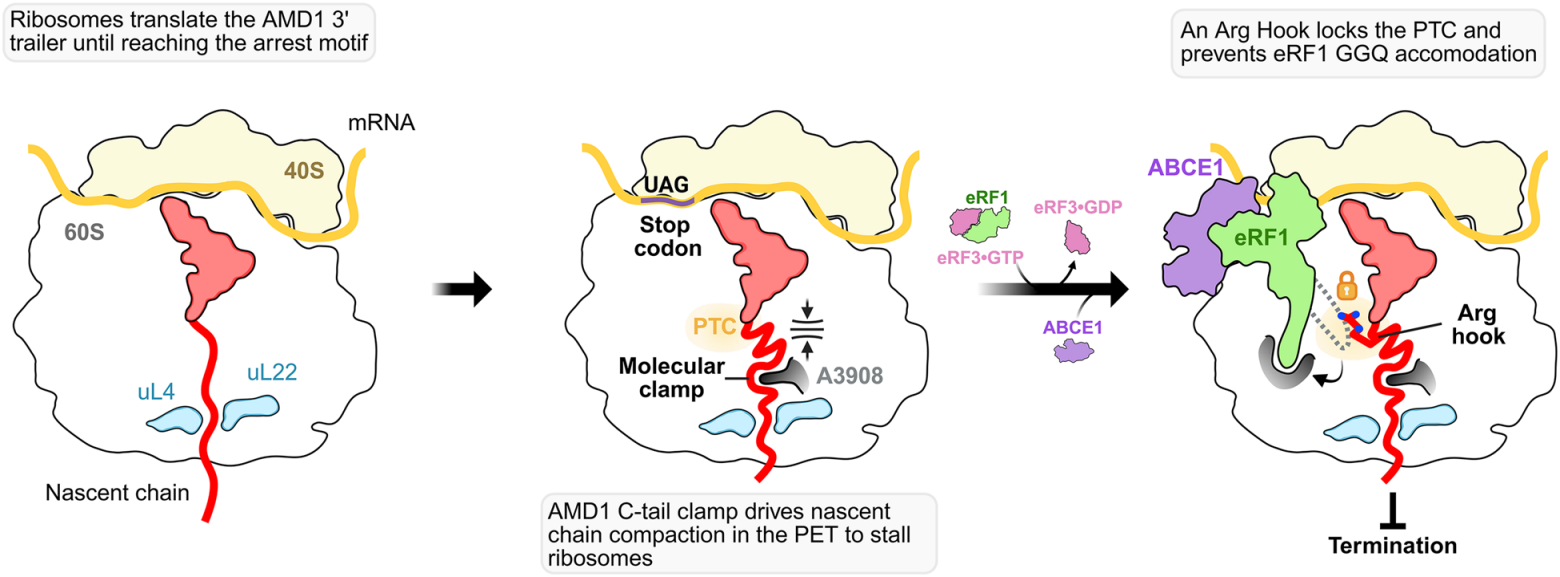
Model for ribosome stalling and termination inhibition by the AMD1 C-tail arrest peptide. Ribosomes extend into the *AMD1* 3′ trailer and pause at a downstream stop codon. The AMD1 C-tail arresting peptide forms a molecular clamp that compacts the nascent chain to stall translation. This clamp helps position an arginine side chain into the PTC, preventing eRF1 GGQ accommodation, thereby blocking translation termination.

In this study, we revealed the molecular mechanism of ribosome stalling and termination inhibition by the AMD1 C-tail arresting peptide, which halts translation at the next in-frame stop codon downstream of the *AMD1* CDS stop codon. We discovered a conserved molecular clamp in the arresting peptide that latches onto rRNA in the exit tunnel to pause translation and drive nascent chain compaction (Fig. 5). This then positions an Arg hook into the PTC altering the architecture required for GGQ loop accommodation. Thus, the AMD1 C-tail has evolved and preserved a precise strategy to lock the PTC and prevent its own release from the ribosome in a two-stage mechanism (Fig. 5).

The PTC contains highly conserved rRNA residues essential for accommodating incoming tRNAs and translation factors on the ribosome (*52*, *56*) and for peptidyl-tRNA hydrolysis (*57*), making it a hotspot for translational arrest. Although mammalian arrest peptides vary in sequence and structure, the few whose structures have been determined, including AMD1, XBP1, and hCMV, stall ribosomes by locally remodeling the PTC through interactions of their C-terminal segments with conserved rRNA nucleotides (fig. S11, A and B). In XBP1 and the current structure of AMD1, a side chain extends into the pocket usually occupied by either the GGQ loop of eRF1, or incoming aminoacyl-tRNAs, disrupting key bases to block termination or elongation, respectively. By contrast, the hCMV peptide adopts a C-terminal helical element whose two terminal prolines shift the nascent chain toward U4531, inducing a ~90° rotation of the base to avoid steric clash (fig. S11C). Despite these distinct geometries, the three peptides modulate the local chemical environment of the PTC to inhibit peptide-bond formation or hydrolysis. Similar strategies are also observed in bacteria, suggesting a shared principle for translational arrest across domains of life (*1*, *10*, *18*).

The locked PTC state induced by the AMD1 C-tail would be also expected to perturb the positioning of any ligand in the A-site including incoming tRNAs. Notably, substituting the stop codon with a sense codon does not relieve stalling (fig. S4C), indicating that the PTC is locked and inaccessible not only to termination factors. For *AMD1*, the presence of a stop codon immediately following the arresting motif may provide a built-in regulatory safeguard: If ribosomes accumulate on this mRNA, termination would release the nascent chain for degradation, supporting the degron-like properties of the C-tail (*38*).

The exploration of existing ribosome profiling data revealed several human mRNA with peaks of ribosome profiling density within CDS extensions caused by potential stop codon readthrough in addition to what was reported earlier (*36*). The majority of these peaks may well result from translation of suboptimal sequences that have not been adapted to be efficiently translated due to the noncoding nature of these regions. However, we also observed that in addition to *AMD1*, stalling in mRNAs of three human genes (*EEF1A2*, *MAPK10*, and *SACM1L*) also occurs in mouse orthologs. This is accompanied by increased nucleotide conservation shortly upstream of stalling sites, suggesting the existence of purifying evolutionary selection acting on the sequence responsible for stalling induction, arguing for functionality and biological significance of these stalls.

Our examination of the putative stall sites from aggregated ribosome profiling data did not reveal shared primary sequence motifs with the conserved AMD1 C-tail stalling elements. This is consistent with most previously characterized natural arrest peptides, which lack consensus motifs leading to unique interactions in the ribosome that induce stalling (*3*, *5*, *8*–*10*). Notably, residues immediately upstream of the stall sites identified in our ribosome profiling analysis are frequently enriched in bulky and/or charged side chains (fig. S10), which may prevent accommodation of eRF1 during termination or aminoacyl-tRNAs during elongation. However, not all arrest peptides impede accommodation like the AMD1 C-tail. SecM and related bacterial arrest peptides permit A-site tRNA accommodation and instead alter the path of the nascent chain in the tunnel (*4*, *58*). The subtle change in the peptidyl-tRNA geometry relative to the pre-attack state (*56*) likely reduces productive peptide-bond formation and leads to stalling. In addition, many stalling peptides may rely on compaction or specific interactions between N-terminal residues in the exit tunnel beyond the stall site similar to the FRK clamp in the AMD1 C-tail (*3*, *59*–*61*). Whether these putative stalling sequences induce short- or long-lived pauses, require trans-acting factors, or act in a cis-acting manner to induce stalling similar to the AMD1 C-tail remains to be investigated.

The mechanism of PTC locking that couples ribosome stalling with termination evasion by the conserved AMD1 C-tail arresting peptide adds to the growing list of regulatory arrest peptides, which use distinct strategies to stall translation. What cellular signals trigger ribosome pausing in the 3′ UTR of *AMD1* and how these stalled ribosomes are rescued remain important questions for future studies.

## MATERIALS AND METHODS

### Cloning and plasmid preparation

Constructs used in this study were designed as previously described (*62*). Sequences encoding for *AMD1* [amino acids (AAs) 78 to 127, *CCEIEVHVEKTFYYMKLYNTCESNSIWFMHSVIFLQVSSKIPHRQGFRPH*] and modified *XBP1* (AAs 237 to 260, *DPVPYQPPFLCQWGRHQCAWKPLM*) were obtained as two gBlocks (Integrated DNA Technologies, Belgium). Both sequences included, from 5′ to 3′, a T7 promoter, 3×FLAG epitope, perfect Kozak sequence, flexible linker, and the indicated stalling sequence.

The *AMD1* gBlock was cloned into a polymerase chain reaction (PCR)–linearized pUC19 vector using Gibson assembly (*63*) with a home-made mastermix. Site-specific mutagenesis was performed using in vivo assembly (IVA) cloning (*64*), in which all candidate codons were substituted with GCG (alanine). All PCR amplifications were carried out with Q5 High-Fidelity DNA Polymerase (New England Biolabs, catalog no. M0491L). For IVA cloning, Dpn I (New England Biolabs, catalog no. R0176L) digestion was performed to remove parental DNA templates.

All constructs were transformed into *Escherichia coli* DH5α competent cells and purified using the GeneJET Plasmid Miniprep Kit (Thermo Fisher Scientific, catalog no. K0503). Correct sequences were confirmed by Sanger sequencing (Eurofins Genomics, Germany).

### In vitro transcription

PRC fragments encoding a T7 promoter and a 3X-FLAG tag linked to the AMD1 C-tail or XBP1 were in vitro transcribed for 4 hours at 38°C with T7 polymerase (1.1 mg/ml) in reaction buffer [40 mM tris-HCl (pH 7.6), 5 mM ribonucleoside triphosphates, 6 mM MgCl_2_, 2 mM spermidine, 1 mM dithiothreitol (DTT), and RNase inhibitor (0.04 U/μl)]. Precipitate was pelleted at 14,000 rpm for 5 min at 4°C. The resulting supernatant was placed in a sterile tube and one volume of 6 M LiCl was added. The mixture was incubated for 1 hour at 4°C and then centrifuged at 14,000 rpm for 20 min at 4°C. The supernatant was discarded, and the resulting pellet was washed with 200 μl of ice-cold 70% ethanol and centrifuged at 14,000 rpm for 5 min at 4°C. The supernatant was discarded, and the pellet was resuspended in 100 μl of sterile ddH_2_O. Resuspended RNA was placed on ice for 5 min and 40 μl of 2.8 M NaOAc and 300 μl of ice-cold 95% ethanol were added. The mixture was incubated on ice for an additional 5 min and then centrifuged at 14,000 rpm for 30 min at 4°C. The resulting pellet was washed with 200 μl of ice-cold 70% ethanol and centrifuged at 14,000 rpm for 5 min at 4°C. After discarding the supernatant, the purified RNA pellet was resuspended in 100 μl of sterile ddH_2_O. The RNA concentration was determined using a nanodrop (Thermo Fisher Scientific).

### In vitro translation reactions for monitoring ribosome stalling

The mRNA corresponding to the AMD1 variants and XBP1 control were in vitro translated in FLEXI rabbit reticulocyte lysate (Promega, catalog no. L4540) diluted to 66.7% (v/v) in buffer [mRNA (0.5 μg/μl), 81 mM KCl, 0.67 mM Mg(OAc)_2_, 24 μM amino acid mix, 0.2 mM spermidine, RNase Inhibitor (0.04 U/μl; Thermo Fisher Scientific), and 0.5× protease inhibitor (Promega)]. The reactions were conducted at 32°C for 25 min and then moved to ice. Samples were diluted in SDS–polyacrylamide gel electrophoresis (PAGE) loading dye [final concentration: 50 mM tris-HCl (pH 6.8), 2% SDS (w/v), 143 mM β-mercaptoethanol, 6% glycerol (v/v), and 0.004% bromophenol blue (w/v)] and loaded on a 4 to 12% bis-tris gel (Genescript, catalog no. M00654). The remaining sample was flash-frozen in liquid nitrogen.

Proteins were resolved and then transferred onto a 0.2-μm nitrocellulose membrane (LI-COR, catalog no. 926-31092). Membranes were blocked in 5% milk in 1× PBST (phosphate-buffered saline with 0.1% Tween-20) for 1 hour at room temperature, with shaking. The membranes were incubated with primary antibodies diluted in 1% bovine serum albumin (BSA) in 1× PBST for 1 hour at room temperature or overnight at 4°C, with shaking. Primary antibodies were as follows: α-FLAG (Sigma-Aldrich, catalog no. F1804, 1:3000) and α-uL1 (Invitrogen, catalog no. MA5-44710, 1:2000). Membranes were incubated for 1 hour at room temperature with secondary antibodies diluted in 5% milk in 1× PBST. Secondary antibodies used: α-mouse (Invitrogen, catalog no. A21058, 1:10,000) and α-rabbit (Invitrogen, catalog no. A32735, 1:10,000). Membranes were imaged with the LI-COR Odyssey imager.

### Alanine scanning mutagenesis experiments

Mutant variants of the AMD1 C-tail were PCR-amplified and mRNA was in vitro transcribed and purified using the RNA Clean & Concentrator Kit (Zymo, catalog no. R1016). The resulting mRNA was translated as described above or using a homemade rabbit reticulocyte lysate as previously described (*62*). In brief, 20 μl of rabbit reticulocyte lysate reactions were prepared. Each translation reaction was split into two equal aliquots: one was treated with RNase A (Promega, catalog no. A7973) for 20 min at 30°C, while the untreated was left in ice. From each aliquot, 1 or 2 μl was diluted in SDS-PAGE loading dye [final concentration: 125 mM tris-HCl (pH 6.8), 4% SDS (w/v), β-mercaptoethanol 10% (v/v), 20% glycerol (v/v), and 0.004% bromophenol blue (w/v)], loaded into a Bolt bis-tris Plus 4 to 12% gradient mini gels, WedgeWell format (Invitrogen, catalog no. NW04127BOX), and run for 23 min. Proteins were transferred to nitrocellulose membranes for 7 min using a Trans-Blot Turbo Transfer System (Bio-Rad). Membranes were blocked in 5% BSA in PBST for 1 hour at room temperature, followed by incubation with primary antibody anti-FLAG (Sigma-Aldrich, catalog no. F1804-200UG; 1:1000 in 1% BSA in PBST) overnight at 4°C with gentle shaking. After washing, membranes were incubated with secondary antibody IRDye 800CW Donkey anti-Mouse IgG (H + L) (catalog no. 926-32212; 1:20,000 in 1% BSA in PBST) for 30 min at room temperature. Signals were visualized using a LI-COR Odyssey imaging system.

### RNC_AMD1C_ purification

RNCs were purified as previously described (*65*). mRNA was in vitro translated using rabbit reticulocyte lysate (Promega, catalog no. L4540) diluted to 66.7% (v/v) in buffer [mRNA (0.5 μg/μl), 81 mM KCl, 0.67 mM Mg(OAc)_2_, 24 μM amino acid mix, 0.2 mM spermidine, RNase inhibitor (0.04 U/μl; Thermo Fisher Scientific), and 0.5× protease inhibitor (Promega)] at 32°C for 25 min and then moved to 4°C. The reaction was incubated with a 200 μl slurry of anti-DYKDDDK beads (Sigma-Aldrich, catalog no. A2220) for 2 hours at 4°C with rotation in a gravity column. Following incubation, the beads were pooled to the bottom of the column using 650 μl of high-salt wash buffer [50 mM HEPES-KOH (pH 7.7), 750 mM KOAc, 10 mM Mg(OAc)_2_, 1 mM DTT, and 0.1% Triton-X]. The column was washed with 10 bead volumes of high-salt wash buffer twice followed by two washes with 10 bead volumes of low-salt wash buffer [50 mM HEPES-KOH (pH 7.7), 100 mM KCl, and 10 mM MgCl_2_]. Elutions were carried out at room temperature with 110 μl of FLAG peptide (0.25 mg/ml) in ribosome buffer [50 mM HEPES-KOH (pH 7.7), 100 mM KOAc, and 15 mM Mg(OAc)_2_] for 15 min (5×). Eluted fractions were placed on ice, combined, and then centrifuged at 100,000 rpm for 1 hour at 4°C using the TLA120.1 rotor (Beckman). The supernatant was discarded, and the pellet was resuspended in ribosome buffer [50 mM HEPES-KOH (pH 7.7), 100 mM KOAc, and 15 mM Mg(OAc)_2_] using a micropipette. The concentration of the purified ribosomes was determined with the nanodrop.

### Cryo-EM sample preparation and data acquisition

Immediately following purification, RNCs were diluted to ~250 ng/μl in ribosome buffer and placed on ice. Mica sheets were coated with carbon using a CCU-010 high-vacuum coating system (Safematic). Sheets with a 3.3-nm carbon layer were floated on sterile ultrapure water, and the carbon film was picked up with Quantifoil R2/1 300 mesh copper grids (Quantifoil, catalog no. Q350CR1). The carbon coated grids were glow discharged at 15 mA for 15 s (EMS). A 5-μl sample of the purified RNCs was incubated on the grid for 1 min at 4°C and 100% relative humidity. Following the incubation period, grids were blotted using a Vitrobot Mark IV (Thermo Fisher Scientific) at a blotting force of 7 and then plunged into liquid ethane cooled to liquid nitrogen temperature. Data were collected at the University of Virginia Molecular Electron Microscopy core using the Thermo Fisher Scientific Krios electron microscope operated at 300 kV and equipped with a K3 direct electron detector at a nominal magnification of 105,000× (pixel size of 0.83 Å), with an energy filter slit width of 10 eV. Data were collected at a defocus range of −1.8 to −0.8 μm with a step size of 0.2 μm. A dose of 50 e/Å^2^ was applied across 40 frames per movie and a total of 11,162 movies were collected.

### Single particle cryo-EM data processing

Cryo-EM data were processed using CryoSPARC version 4.6.0 (*66*). Movies were patch motion corrected and subjected to patch CTF estimation. Particles with minimum and maximum diameters of 250 and 400 Å, respectively, were selected using blob picker. Particles were extracted at a box size of 464 × 464 pixels and binned to 128 pixels for initial 2D and 3D classifications. The 173,341 particles from the 2D classification showing ribosome classes were used for ab initio reconstruction to generate initial models for subsequent heterogeneous refinement to remove rotated and collided ribosomes. The particles were then re-extracted at full box size without binning and refined to generate a consensus ribosome structure at 2.76 Å. Iterative 3D variability analyses (*67*) were conducted to sort for a homogenous stack of particles containing high resolution features for the additional factors observed in the consensus structure. Focused masks were used encompassing the A- and P-site of the ribosome. Following three rounds of 3D variability analyses, a final set of 15,310 particles containing the bound factors were refined to an average resolution of 2.86 Å determined by the gold-standard Fourier shell correlation (FSC) with an FSC cutoff of 0.143. The local resolution was calculated in CryoSPARC at an FSC threshold of 0.143.

### Model building

A previously published model of the 80S ribosome [Protein Data Bank (PDB) 6R5Q], eRF1 (PDB 5LZU), and ABCE1 (PDB 3JAH) were fitted into the final cryo-EM map using ChimeraX (*68*). Individual domains and helices of eRF1 and ABCE1 were further adjusted as rigid bodies in Coot version 0.9.6 (*69*). Models were manually adjusted on the basis of observed side chain densities in the EM map. The AMD1 C-tail arresting peptide atomic model was built de novo based on the observed side chain densities using available sequence information. Because of the lack of a reliable sequence for the mammalian tRNA-His (AUG), the tRNA-His sequence from *Danio rerio* (tRNA-His-ATG-3-1 from GtRNAdb) was used to model the P-site tRNA. This sequence largely matched the EM density with one A/U and G/C base swap. The final model was iteratively refined in PHENIX version 1.20.1-4487 using three macrocycles of real space refinements applying Ramachandran, sidechain rotamer, protein secondary structure, and nucleotide restraints (*70*). Molprobity was used to validate the model and resolve clashes. All cryo-EM figures and models were made in ChimeraX (*68*).

### Ribosome profiling data analysis

For ribosome profiling data analysis, we used 3835 human libraries from RiboSeq Data Portal (*39*) mapped to the human genome in RiboCrypt. For the analysis of *AMD1* ortholog, species supported by RiboCrypt with at least 3 ribosome profiling libraries were used to generate *AMD1* transcript-level ribosome occupancy plots (fig. S1). For the analysis of pausing at the readthrough extensions, we have included all cases annotated as readthrough according to GENCODE version 47 catalog except for *AGO1*, *VEGFA*, and *ACP2* that are lacking ribosome profiling support for stop codon readthrough in RiboCrypt upon manual examination. In addition, we identified candidate stop codon readthrough genes based on the following parameters using ORFik R library (*71*) (extension length >6 codons, ORFScore >5, in-frame RPF reads >300, codons covered >3, codons with in-frame reads >10%, and in-frame versus out-of-frame read ratio >50%). Subsequently any cases where readthrough extensions overlap annotated CDS from other transcripts have been excluded. The resulting 78 readthrough candidates have been examined for the presence of outlier peaks of ribosome profiling density at individual positions. A peak of read density was considered to be an outlier if its density *Z* score >5. Subsequently the profiles containing such peaks have been examined manually. For the 12 cases with confirmed outlier peaks, we have analyzed nucleotide conservation using PhyloP. We also have examined ribosome profiles of mouse orthologs for these candidates. To constructs sequence logos shown in fig. S10, for each locus of interest we have extracted segments of 100-way vertebrate genomic alignment (*72*) using CodAlignView. The nucleotide sequences surrounding the region upstream of the stall sites were conceptually translated in the reading frame matching that of CDS using the standard genetic code (table 1 in https://ncbi.nlm.nih.gov/Taxonomy/Utils/wprintgc.cgi) and realigned using Clustal Omega (1.2.4) multiple sequence alignment (*73*). Sequence logos were generated from these alignments with WebLogo 3 (*74*).
