# Supplementary material for: Mechanism of ribosome stalling by the AMD1 C-terminal tail arrest peptide
Source: Sci Adv. 2026 Mar 27;12(13):eaec5067. doi: 10.1126/sciadv.aec5067 (PMC13025110; doi:10.1126/sciadv.aec5067)
Supplement: Supplementary file 1 — Figs. S1 to S11 Table S1 Legends for movies S1 to S3 [file sciadv.aec5067_sm.pdf]

Science Advances
AAAS

Supplementary Materials for

Mechanism of ribosome stalling by the AMD1 C-terminal tail arrest peptide

Emir Maldosevic *et al.*

Corresponding author: Pavel V. Baranov, p.baranov@ucc.ie; Ahmad Jomaa, ahmadjomaa@virginia.edu

Sci. Adv. **12**, eaec5067 (2026)
DOI: 10.1126/sciadv.aec5067

The PDF file includes:

Figs. S1 to S11
Table S1
Legends for movies S1 to S3

Other Supplementary Material for this manuscript includes the following:

Movies S1 to S3

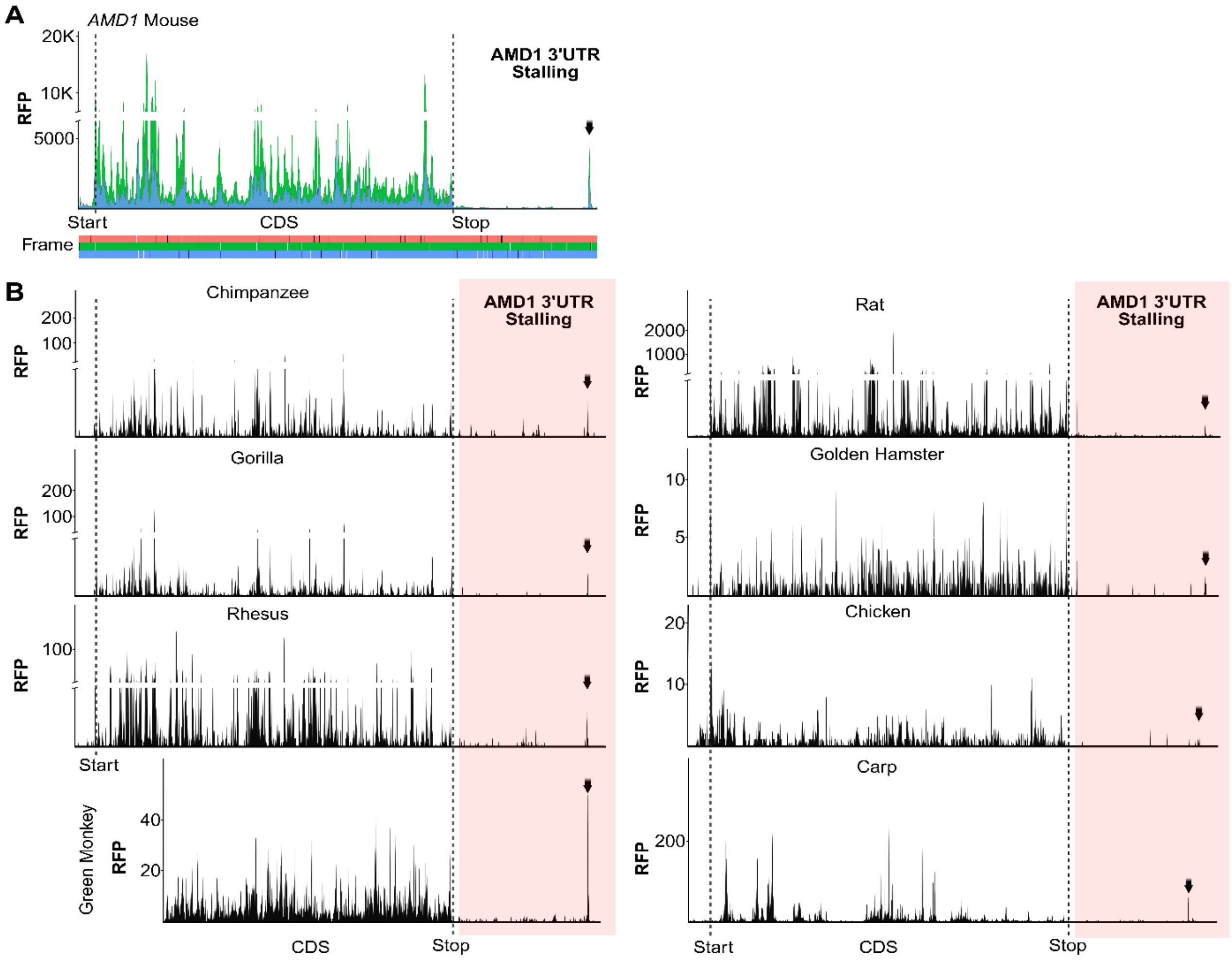

Fig. S1

***AMD1* ribosome profiles of vertebrate homologs.** A) Aggregated ribosome sequencing reads for the mouse *AMD1* transcript (ENSMUST00000099945) showing the CDS and a part downstream of it that contains the sight of ribosome stalling. B) Ribosome profiles of *AMD1* mRNAs for the indicated vertebrates highlighting peaks present at the end of *AMD1* extended translon (red). The green monkey (*Chlorocebus sabaeus*) ENSEMBL transcript ENSCSAT00000013631.1 is truncated according to our examination of the genomic region and is missing two 5' exons.

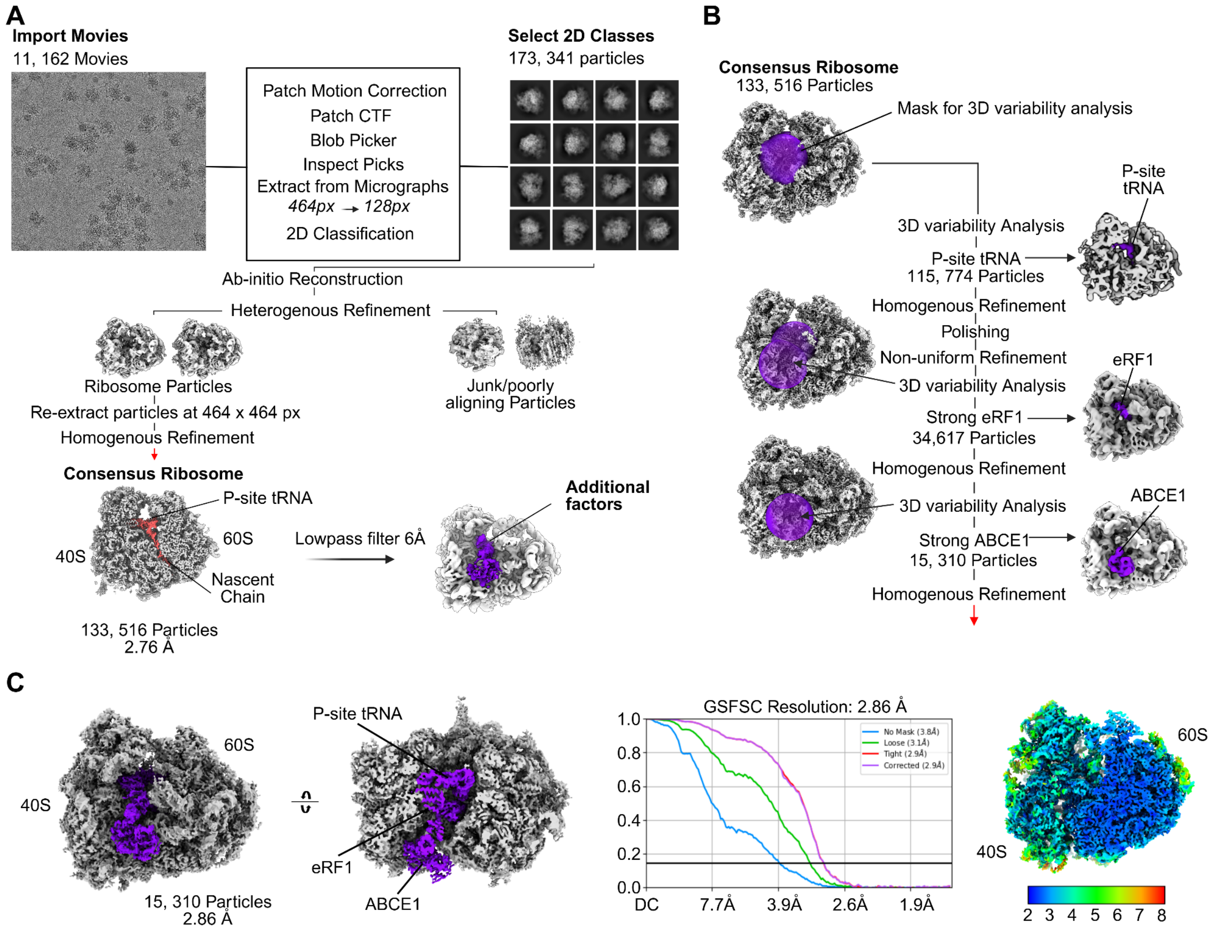

Fig. S2

Single particle cryo-EM processing workflow used to determine the ABCE1:eRF1:RNC$_{\mathbf{AMD1C}}$ structure. A) Movies were patch motion corrected and CTF estimated in CryoSPARC. Blob picker was used to select for particles from the resulting corrected micrographs. Particles were extracted at a box size of 464 x 464 pixels and binned to 128 pixels. Following 2D classification, 173, 341 particles were selected and used to generate ab-initio models. The models were used in subsequent heterogenous refinements to sort for ribosome particles and discard junk or poorly aligning particles. Consensus ribosomes were homogeneously refined and displayed strong P-site and nascent chain density (red) along with additional density for factors interacting at the A-site of the ribosome (purple). B) The consensus ribosome particles

were iteratively classified by 3D variability analysis using the indicated focused masks (purple spherical mask) to reach a homogenous subset of ribosome particles containing P-site tRNA, eRF1 and ABCE1 (purple). C) The final eRF1:ABCE1:RNC_{AMD1C} particles were homogeneously refined to an average resolution of 2.86 Å, determined by gold standard Fourier Shell Correlation (GSFSC) at an FSC cutoff of 0.143. The local resolution was estimated in cryoSPARC at an FSC threshold of 0.143.

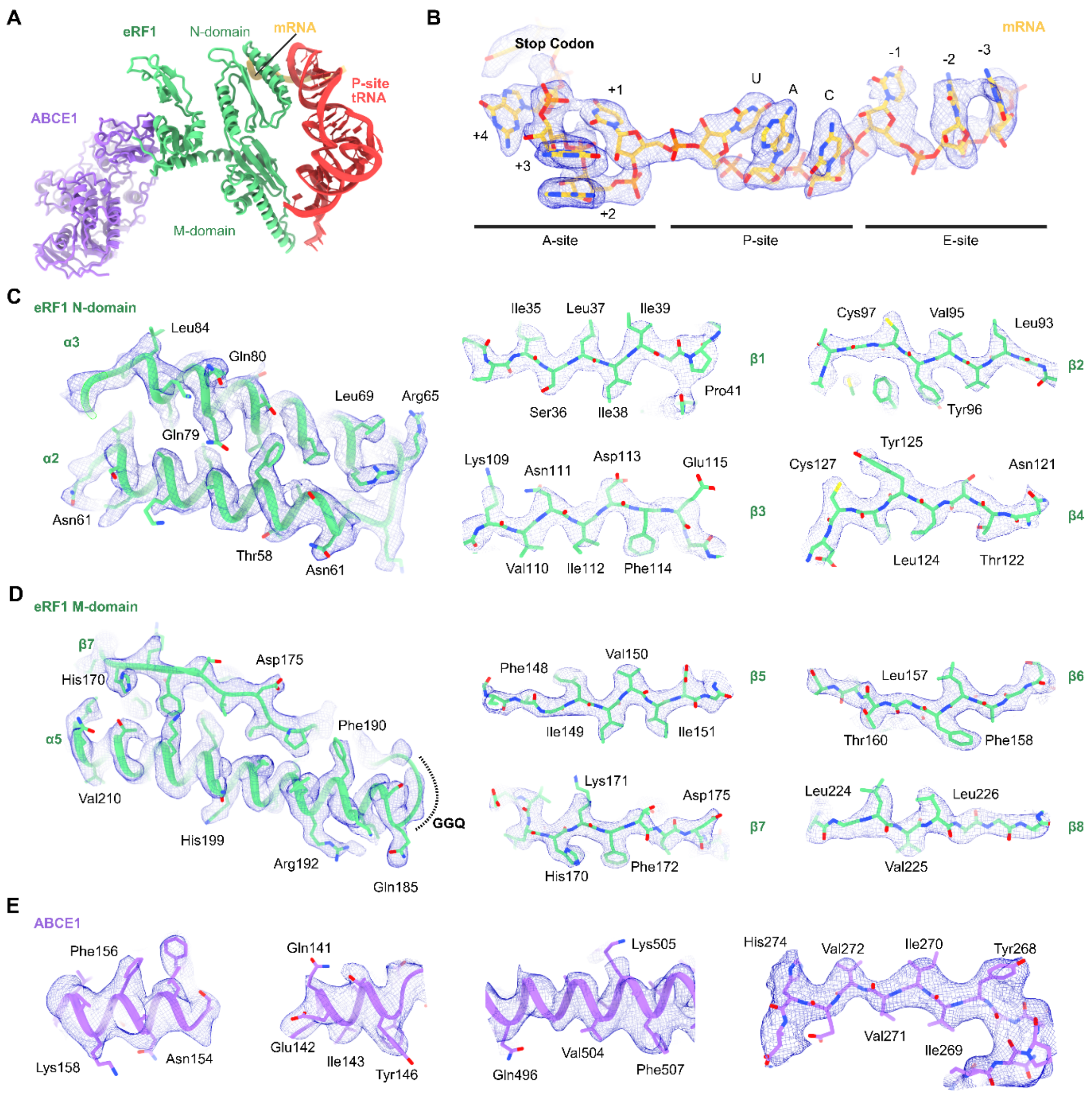

Fig. S3

Structure of ABCE1 and eRF1 in complex with a P-site tRNA and mRNA on the stalled ribosome. A) Overview of the ABCE1, eRF1, mRNA, and P-site tRNA models shown without the rest of the ribosome for clarity. B-E) Fits for the indicated model regions of the mRNA, eRF1 N- and M-domains, and ABCE1. Sharpened EM maps (b-factor -29.0Å^2) are used to visualize the mRNA and eRF1. Unsharpened maps were used for visualizing ABCE1.

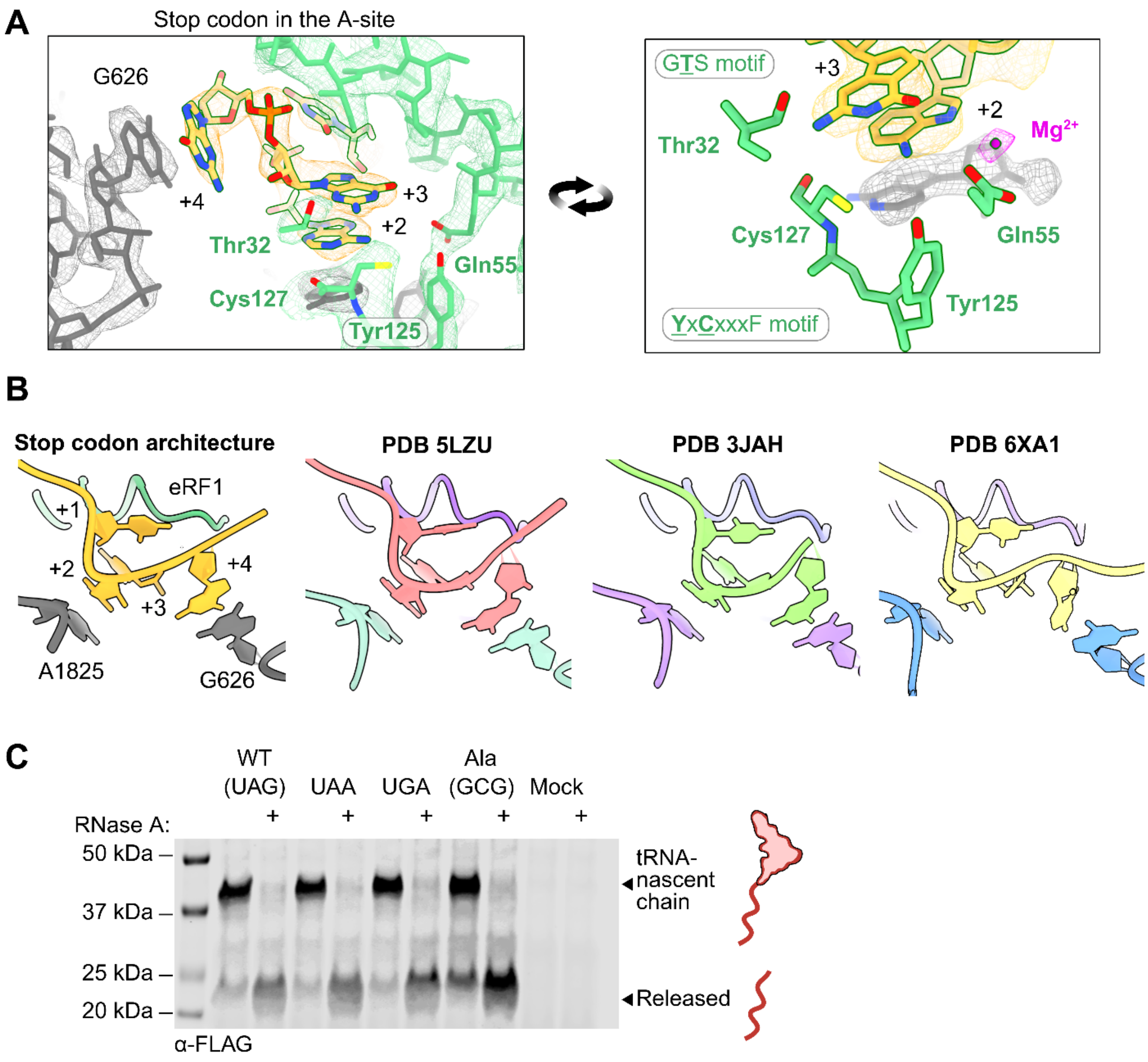

Fig. S4

Stop codon recognition in the A-site by eRF1 does not affect ribosome stalling. A) Closeups of the stop codon interactions with the N-domain motifs of eRF1. Sharpened maps (b-factor -29.0Å^2) were used for visualization. B) Comparison of stop codon compaction following recognition by eRF1 with other termination complexes (PDB 5LZU, 3JAH, 6XA1). C) Mutations of the native stop codon in the AMD1 C-tail.

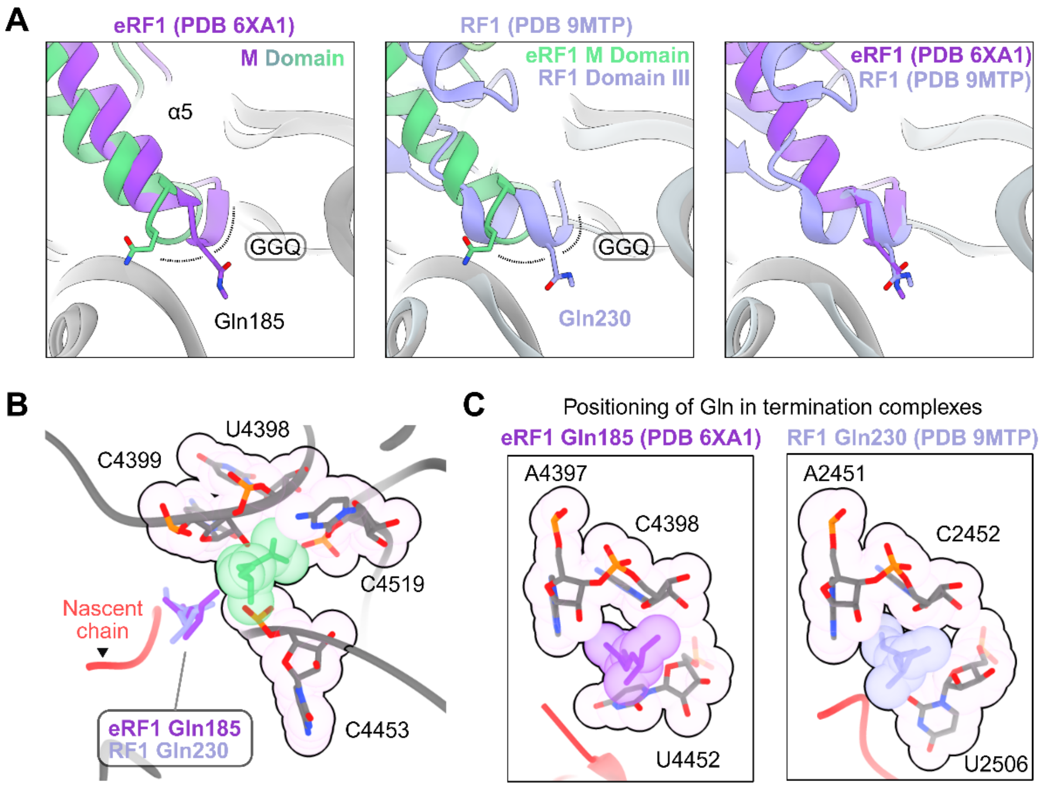

Fig. S5

Comparison of GGQ motif in AMD C-tail stalled ribosome versus other termination complexes. A) Comparison of GGQ positioning in the current structure (green) and other termination complexes (purple) of eRF1 and RF1. B) Sequestration of the eRF1 Gln185 (green) on ribosomes stalled by the AMD1 C-tail in a pocket away from the PTC compared to other termination complexes (purple). C) Closeups of the proper positioning of glutamines that make up the GGQ motif at the PTC in other termination complexes. Models used for comparison: PDB 6XA1 (human eRF1), PDB 9MTP (bacterial RF1).

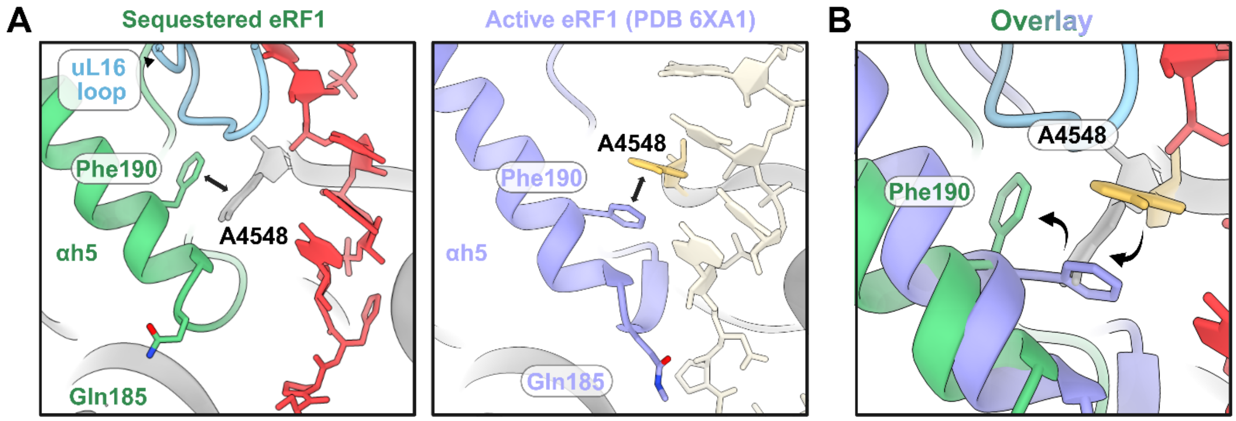

Fig. S6

A conformational change in eRF1 αh5 and uL16 interactions observed in the AMD C-tail stalled ribosome. A) The flexible loop of uL16 was resolved interacting with the P-site tRNA. eRF1 Phe190 π-stacks with A4548 in a distinct conformation when sequestered. B) Closeup of the conformational changes in Phe190 and A4548 relative to the previous termination complex (PDB 6XA1).

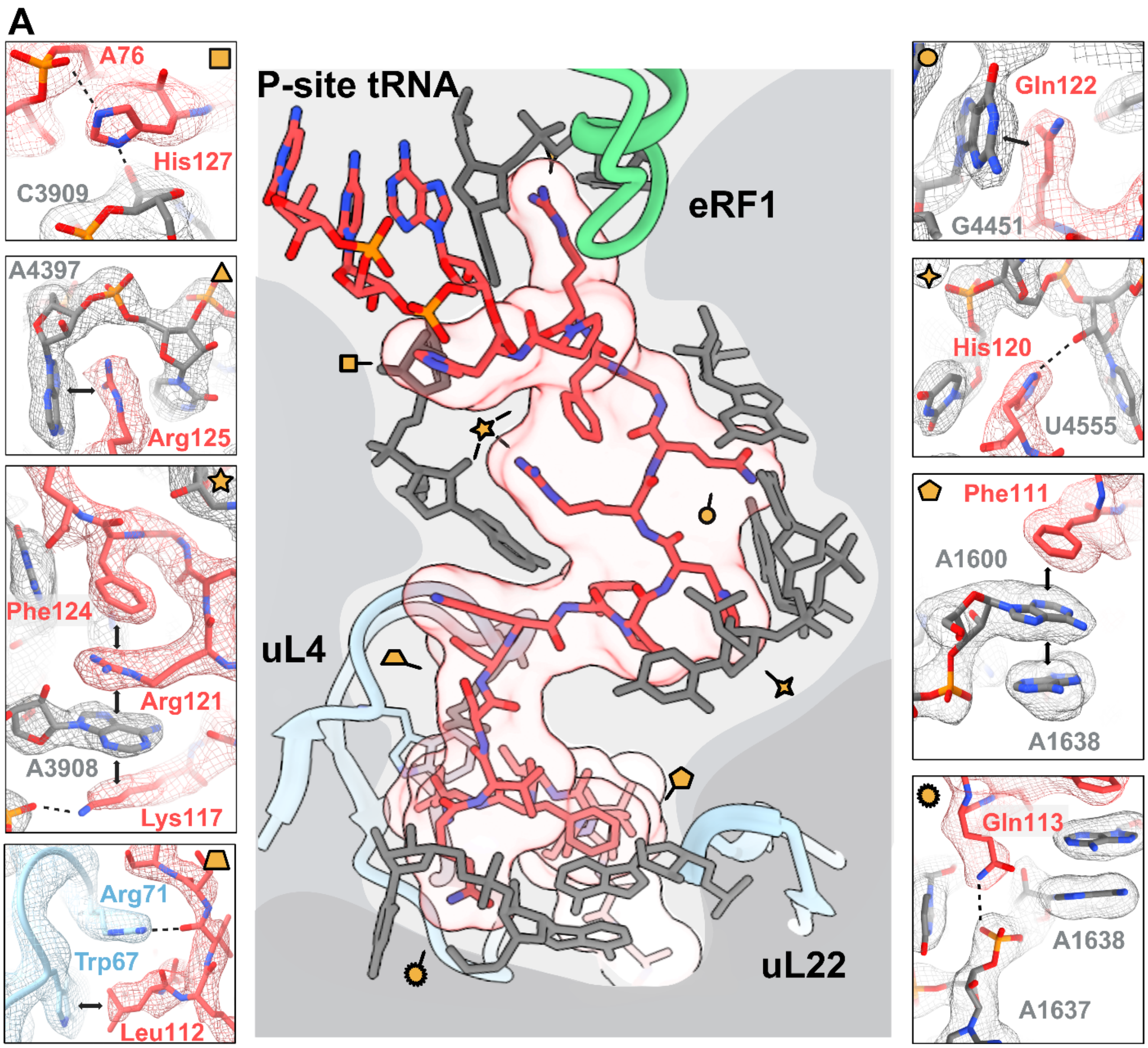

Fig. S7

The AMD1 C-tail stalls the ribosome via an extensive interaction network in the exit tunnel.

A) Central panel depicts a model of the nascent chain shown as a transparent surface flanked by interacting bases and ribosomal proteins from the PET. From the top left: hydrogen bond between His127 and the backbone of A76 and C3909; π-stacking interaction between Arg125 and A4397; π-stacking network formed between Phe124, Arg121, A3908, and Lys117; hydrogen bond formed between Arg71 (uL4) with the carbonyl oxygen of Val114 and hydrophobic interaction between

Trp67 (uL4) and Leu112. From the top right: the AMD1 C-tail forms an additional stacking interaction between Gln122 and G4451; polar interaction between His120 and U4555; π-stacking interaction between Phe111, A1600, and A1638; polar interaction between Gln113 and A1638 backbone. Sharpened maps (b-factor -29.0Å^2) are depicted as mesh for visualization.

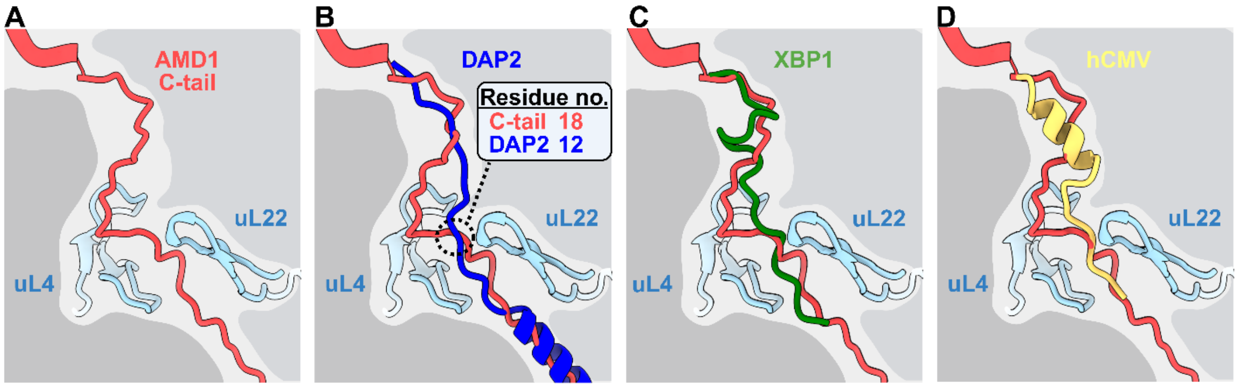

Fig. S8

Comparison of nascent chains in the exit tunnel of mammalian arresting peptides and the non-arresting peptide DAP2 in the PET. A) The AMD1 C-tail adopts a unique Z-shaped configuration in the exit tunnel leading to the constriction site formed by uL22 and uL4. B) DAP2 does not display any notable compaction or secondary structural features when compared to the AMD1 C-tail. The number of residues from the C terminus to the constriction site is listed for AMD1 C-tail and DAP2. C) XBP1 adopts a short, compacted S-shaped configuration when compared to the AMD1 C-tail. D) The hCMV arresting peptide forms a short α-helix to stall ribosomes while the AMD1 C-tail does not form any secondary structures prior to the constriction site. Models used for comparison: DAP2 (PDB 7OBR), XBP1 (PDB 6R5Q), hCMV (PDB 5A8L).

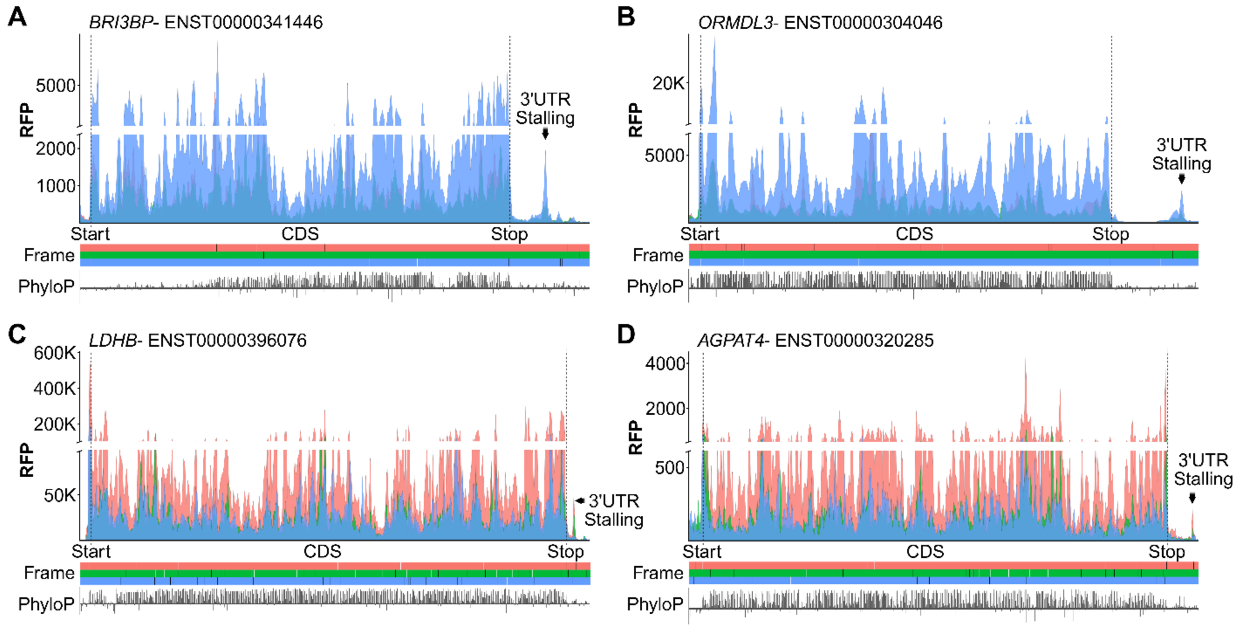

Fig. S9

Representative ribosome profiles for the indicated human genes with stalling present in the 3'UTR. Aggregated human sequencing reads for *BRI3BP* (A), *ORMDL3* (B), *LDHB* (C), and *AGPAT4* (D). Plots were generated with ribocrypt.org and show the CDS region with potential stop codon readthrough regions containing stalling sites as indicated.

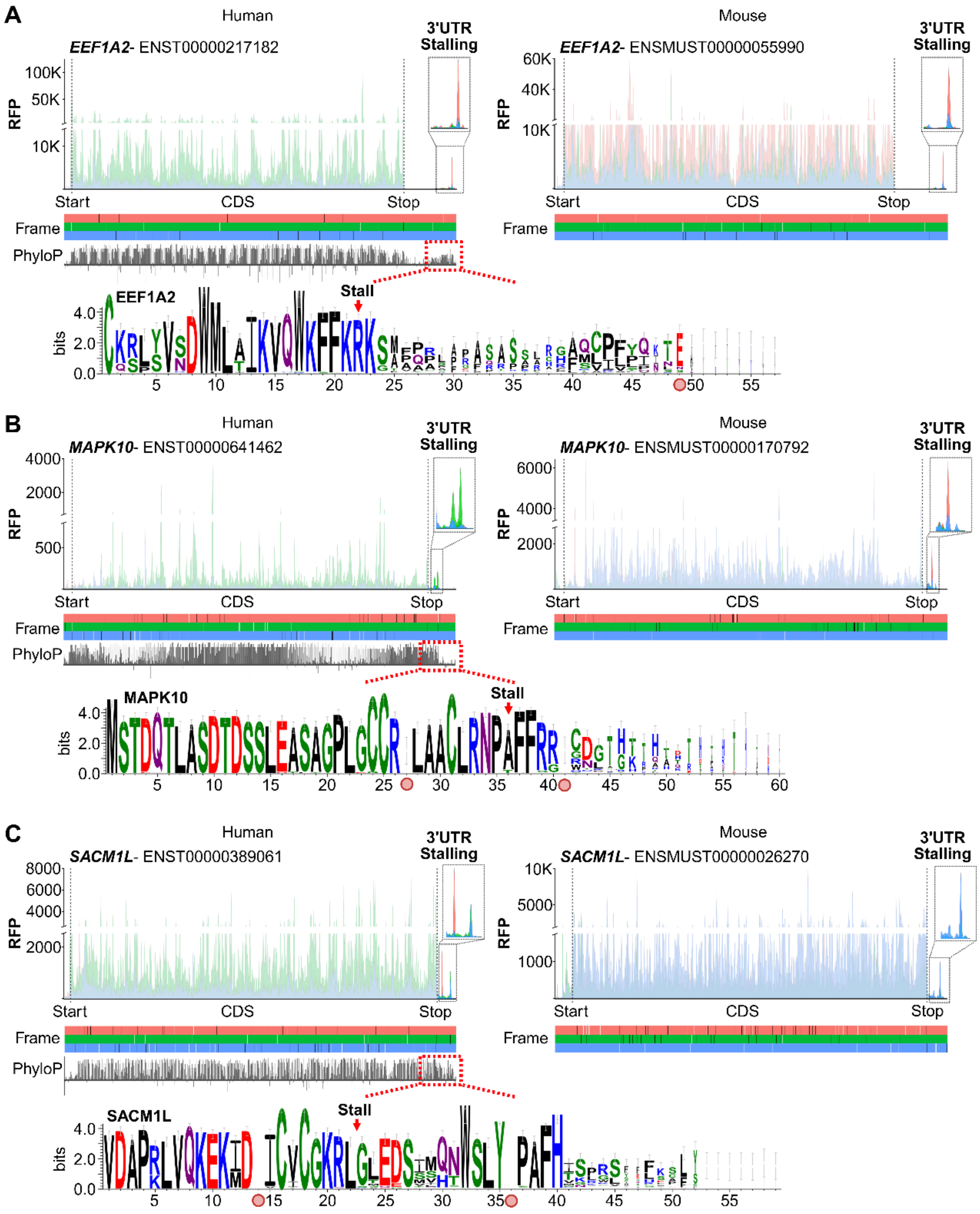

Fig. S10

Ribosome stalling in 3' trailers of select candidates present in mice ribosome sequencing data. A) Ribosome profile of human (left) and mouse (right) *EEF1A2*. B) Ribosome profile of human (left) and mouse (right) *MAPK10*. C) Ribosome profile of human (left) and mouse (right)

SACM1L. All plots are aggregated data of ribosome sequencing reads from multiple studies generated with ribocrypt.org showing the CDS region (transparent) with indicated stalling sites at the end of extended translons. Sequence logos below each ribosome profile depict conservation in the region surrounding the stall site (red boxed area). Red arrows indicate stall sites on the sequence logo plots. Red circles indicate stop codons in the human sequence at the indicated position on the x-axis.

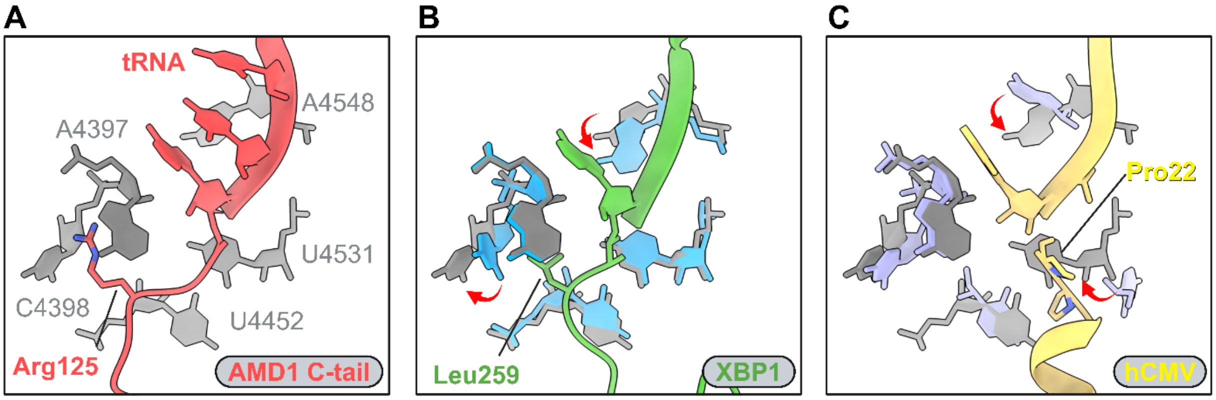

Fig. S11

Positioning of key PTC bases in mammalian arrest peptide structures. A-C) Close-up of the AMD1 C-tail, XBP1 (PDB 6R5Q) and hCMV uORF (PDB 5A8L) arrest peptides at the PTC. Arrows depict conformational changes observed for rRNA bases relative to the AMD1 C-tail model.

	**eRF1:ABCE1:AMD1$_{C\text{-tail}}$**
EMDB code	EMD-72314
PDB code	PDB-9Q7Q
**Data collection and processing**	
Nominal magnification	105,000x
Voltage (kV)	300
Electron exposure ($e^{-}/Å^2$)	50
Defocus range (μm)	-1.8/-0.8
Pixel size (Å)	0.83
Initial particle images (no.)	133, 516
Final particle images (no.)	15, 310
Map resolution at FSC=0.143 (Å)	2.86
**Structure refinement in PHENIX 1.20.1**	
Model resolution at FSC=0.5 (Å)	3.1
CC_{mask}	0.80
Map sharpening B factor ($Å^2$)	-29
Model composition	
Non-hydrogen atoms	218503
Protein residues	12612
RNA residues	5464
B factors min/max/mean ($Å^2$)	
Protein	0.00/109.15/52.60
RNA	0.00/165.06/63.42
Ligand	0.00/125.13/27.89
RMSD	
Bond lengths (Å)	0.003
Bond angles (°)	0.665
**Validation**	
MolProbity score	1.65
Clashscore	8.56
Poor rotamers (%)	1.09
Ramachandran plot	
Favored (%)	0.08
Allowed (%)	2.85
Outliers (%)	97.07
Validation (RNA)	
Good sugar pucker (%)	99.27
Good backbone (%)	80.14

Table S1
Cryo-EM data collection, model refinement and validation statistics.

Movie S1
Overview of the ABCE1-eRF1-RNC$_{AMD1C}$ complex.

Movie S2
The AMD1 C-tail clamp mediates arrest peptide compaction in the PET.

Movie S3
An arginine hook within the AMD1 C-tail inserts into the PTC to block eRF1 Gln185 accommodation.
